# Allies in the Skin Defense System: The Role of Thread Cells in the Evolution of Hagfish (Myxiniformes)

**DOI:** 10.3390/biology14121662

**Published:** 2025-11-24

**Authors:** Sebastian Marino, Alessio Alesci

**Affiliations:** 1 Department of Chemical, Biological, Pharmaceutical and Environmental Sciences, University of Messina, Viale Stagno d’Alcontres, 31, 98166 Messina, Italy; sebastian.marino@iusspavia.it; 2 Science, Technology and Society Class, University School for Advanced Studies IUSS, 27100 Pavia, Italy

**Keywords:** thread cells, hagfish, defense, skin

## Abstract

The skin of vertebrates serves as a crucial interface with the external environment. In fish, it performs various functions, mainly offering protection against pathogens through the action of specialized cells. Cyclostomes, such as hagfish, lack scales and rely heavily on mucus for defense. These jawless vertebrates possess specialized glands that produce a unique mucous exudate when threatened, forming a thick slime that can clog the gills of predators. This substance, composed of mucus and filamentous proteins, offers hagfish a distinct evolutionary advantage and may explain their survival among extinct agnates. These proteins are produced in the cytoplasm of epidermal thread cells, which are unique to hagfish and contain coiled, intermediate filaments. Despite extensive research on thread cell morphology, their roles remain poorly understood. This study investigates the putative defense function of epidermal thread cells in three hagfish species, *Eptatretus cirrhatus* (J. R. Forster, 1801), *Eptatretus stoutii* (Lockington, 1878), and *Myxine glutinosa* (Linnaeus, 1758), using immunohistochemistry, confocal microscopy, and bioinformatics techniques to better understand their contribution to hagfish immunity and ecological resilience.

## Simple Summary

The skin of vertebrates protects them from external factors. In fish, it mainly defends against microbes using specialized cells. Hagfish, jawless vertebrates that do not possess scales, use mucus as their primary defense. When in danger, they release a thick slime made of mucus and filamentous proteins that can obstruct the gills of the predators. These filaments are produced by thread cells, which are peculiar skin cells found only in hagfish. Although their structure has been well studied, their involvement in defense mechanisms remains unclear. This study investigates the possible involvement of these cells in the defense mechanisms of hagfishes using immunohistochemistry, confocal microscopy, and bioinformatic techniques to learn more about their immune system and survival.

## 1. Introduction

The Myxinidae family comprises 82 species of hagfish, with new species being discovered worldwide at an increasing rate [1]. As suggested by genetic evidence, hagfish, together with lampreys, form the Cyclostomi superclass [2]. Opposed to fish, cyclostomes have smooth, scale-free skin covering their bodies [3]. They possess several unique features, such as circular mouths that lack jaws, the absence of paired fins, and corneous epidermal structures that function as teeth, called ceratodontes [4]. In these organisms, and mainly in myxines, protection depends heavily on mucus secretion from the skin [4]. When threatened, hagfish use a special defense mechanism that involves the rapid release of a mucous exudate from a specialized slime structure, which can obstruct the gills of predators, impeding their breathing [5]. Hagfish extruded slime contains protein threads that unravel upon contact with seawater, creating a thick, slimy substance [6]. Zintzen et al. (2011) proposed that hagfish extruded slime has several functions, including preventing gill-breathing species from preying on them, reducing competition for food by keeping out other scavengers, and possibly even choking their prey [5]. This special slimy exudate offers a remarkable advantage to hagfishes against gill-breathing predators and potentially demonstrates how hagfishes have endured while almost every other jawless vertebrate has gone extinct since the rise in the gnathostomes [7]. This substance is produced in epidermal structures located on the ventrolateral sides of the hagfish, which are composed of large amounts of two secretory cells, the thread and mucous cells [8].

Thread cells are unique to hagfish and contain a highly condensed coil of intermediate filaments in their cytoplasm in the form of a single fiber [9]. These threads contain α- and γ-fibrous proteins that are part of the intermediate filament family [10]. These fibers assemble in the apical region of the cell nucleus, and their elongation follows a loop pattern, forming a conical coiled structure in mature thread cells [11]. When the slime structures are triggered to release their contents by a mechanical stimulus, both thread and mucous cells are activated [12]. Holocrine secretion causes the rupture of the plasmatic membrane of these cells, resulting in the release of intact mucin vesicles and threads into seawater [6,13]. The interaction between mucins and threads allows the formation of a thick structure capable of trapping large amounts of water in a short period [13]. Thread cells also contain large amounts of granules in their cytoplasm; however, their role and contents remain unclear [12]. Zeng et al. (2023) proposed different functions for these granules, such as deterring predators by containing distasteful compounds, a defensive role by containing antimicrobial molecules, or an alarm role by containing pheromones [14,15].

In this study, a total of three hagfish specimens have been analyzed: the broadgilled hagfish (*Eptatretus cirrhatus*, J. R. Forster 1801), which can be found around the coasts of New Zealand and Australia at depths ranging between 1 and 1100 m [16]; the Pacific hagfish (*Eptatretus stoutii*, Lockington 1878), which can be found in the Pacific Ocean, where it inhabits clay bottoms at depths ranging from 16 to 966 m [17]; and the Atlantic hagfish (*Myxine glutinosa*, Linnaeus 1758), which lives in the Atlantic Ocean, usually on the muddy ocean floors at depths of approximately 20 to 1000 m [18]. These organisms prefer environments rich in clay, sand, or gravel, which reduce friction on their skin on the substrate [4]. They are also adapted to the hypoxic environment found in muddy bottoms or inside large fish carcasses [19]. Moreover, they possess a scavenger lifestyle, often eviscerating the bodies of dead or injured animals [19]. It has been shown that myxines are able to absorb nutrients from the water, like amino acids, directly through their skin and gill epithelia [20,21].

Hagfish play a crucial role in marine ecosystems, contributing to the health of the seafloor by processing bycatch discards and facilitating organic matter recycling. In recent decades, hagfish have become important to the fishing economy for their skin leather and as a food source [22]. Despite extensive morphological descriptions [9,11,12] and biochemical analyses of the slime components [6,8,13], the functional role of the thread cells in the defense mechanism remains poorly understood. Specifically, the literature has focused primarily on the thread’s structural protein composition and its role in rapid gel formation [11,13]. This study aims to investigate, for the first time, the possible role of epidermal thread cells in the skin innate defense of hagfish, a critical, unexplored knowledge gap, using immunohistochemistry, confocal microscopy, and bioinformatics techniques, evaluating the presence of potential innate immune markers for these cells.

We hypothesize that thread cells are not solely mechanical components but also harbor markers of the innate immune system and may play a direct defensive role, possibly through the release of antimicrobial peptides or other immune factors, recognizing antigens, and stimulating mucus release.

## 2. Materials and Methods

### 2.1. Specimen Collection

Skin samples from the dorsolateral region of trunk of three different species of hagfish (*Eptatretus cirrhatus*, *Eptatretus stoutii*, and *Myxine glutinosa*) were obtained from our laboratory’s histotheca (a collection of long-lasting histological slides) and underwent a processing protocol to create durable preparations for optical microscopy and paraffin block storage. The study included a total of nine specimens (*N* = 3 adult individuals per species), ensuring that observations accounted for inter-individual and inter-specific variability.

### 2.2. Tissue Preparation and Histological Analysis

Skin samples were fixed in 10% formalin solution in 0.1 M phosphate-buffered saline (pH 7.4) for at least 2 h. Afterwards, they were dehydrated in graded ethanol (from 50% up to 100%, 1 h each) and rinsed in xylene before being embedded in Paraplast^®^ (McCormick Scientific LLC, Saint Louis, MO, USA). Serial slices (4–5 μm thick) were cut using a rotary microtome (LEICA 2065 Supercut, Nussloch, Germany). Two sections were placed on each slide, having a total of 10 slides per specimen. The sections were collected and dried at 38 °C for at least 48 h before being deparaffinized and rehydrated in graded ethanol (10 min each for 100%, 95% and 80%, 5 min each for 70%, 50% and 30%). The sections were then stained using Hematoxylin and Eosin (H/E) (05-06002; 05-10007 Bio-Optica Milano S.p.A., Milano, Italy), Alcian Blue pH 2.5-PAS (04-163802 Bio-Optica Milano S.p.A., Italy), and Toluidine blue (05-M23001 Bio-Optica Milano S.p.A., Italy) for optical microscopy evaluation. Finally, the slides were dehydrated in ethanol (from 30% to 100%) and rinsed in xylene. Coverslips were mounted on the slides using Eukitt mounting medium (Sigma-Aldrich, Saint Louis, MO, USA). To obtain photos, an Alexasoft TP3100A CMOS digital camera (Alexasoft; Firenze, Italy), mounted on an optical microscope, was used.

### 2.3. Rationale for Antibody Selection for Thread Cells

The selection of marker proteins is based on their established relevance to the defensive function, signaling mechanism, and structural integrity of the hagfish thread cells. Piscidin-1 was chosen as an antimicrobial peptide indicative of the acute alarm and defense response characteristic of the hagfish slime system [23]. Following this defensive signaling cascade, TLR2 was included as a key pattern recognition receptor potentially involved in immune surveillance and rapid cellular activation upon insult [24]. To characterize the structural components, α-tubulin was selected to assess the microtubule network, crucial for rapid cellular movement and organization during thread extrusion [25]. Muc2 was used as a marker of the associated mucous secretory activity [26], acknowledging the functional synergy between thread cells and the surrounding mucous cells. Finally, Vimentin was chosen to characterize the intermediate filament components [27], which are fundamental to the unique and robust cytoskeletal architecture required for rapid thread assembly. Collectively, these markers enable a comprehensive evaluation of both the core structural elements and the broader defensive and signaling functions of thread cells upon external stimulus. Prior literature also supports the involvement of all tested antibodies in immune and defense functions within related contexts [28,29,30,31,32,33,34].

### 2.4. Immunoperoxidase

The presence of Piscidin-1 (GenScript, Piscataway, NJ, USA, dilution 1:100, source: rabbit) was assessed using immunohistochemical techniques with an optical microscope. All immunoperoxidase assays were performed independently on triplicate sections (three biological replicates per individual) for each of the nine specimens, ensuring the reproducibility of the staining pattern. The slides were deparaffinized in two washes of xylene (10 min each) and rehydrated in graded ethanol (from 100% to 30%). Subsequently, they were treated with 3% hydrogen peroxide for 30 min. The slides were incubated for 60 min at room temperature with goat anti-rabbit IgG-peroxidase conjugate (Sigma-Aldrich, St. Louis, MO, USA; dilution 1:100; source: goat) after being treated with the antibody for an entire night in a humidified chamber. The sections were incubated for 10–15 min at room temperature in a solution containing 0.02% diaminobenzidine (DAB) and 0.015% hydrogen peroxide to measure the peroxidase activity of the sections. Experiments were run without the primary antibodies as a negative control. Afterwards, the slices were rinsed in PBS, dried, mounted, and examined with an Alexasoft TP3100A CMOS digital camera and a Zeiss Axioskop 2 Plus microscope (Zeiss S.p.A., Oberkochen, Germany).

### 2.5. Immunofluorescence and Confocal Microscopy Analysis

To assess the reliability of the results, all immunofluorescence double-labeling experiments were repeated on at least three independent sections per individual (technical replicates) from all nine specimens. The slices were first deparaffinized in two washes of xylene (15 min each) and rehydrated in graded ethanol (10 min each for 100%, 95% and 80%, 5 min each for 70%, 50% and 30%). Subsequently, slides were immersed in a 1% solution of Sodium Borohydride for 20 min, to remove any tissue autofluorescence. Later, a 2.5% bovine serum albumin (BSA) solution was added to the sections for 20 min. Primary antibodies were added in pairs, one monoclonal and one polyclonal (TLR2/Muc2; Vimentin-/Muc2; Vimentin/α-tubulin) to the sections simultaneously, which were then placed at 4 °C in a humid chamber overnight. The next day, after the slides were rinsed in PBS (two rinses, 5 min each), they were incubated for 30 min with secondary antibodies: an Alexa Fluor donkey anti-rabbit IgG TRITC conjugate and an Alexa Fluor 488 donkey anti-mouse IgG FITC conjugate (Molecular Probes, Invitrogen, Eugene, OR, USA, 1:300). The slides were incubated for 30 min with the secondary antibody. Experiments were run without the primary antibodies as a negative control, even to exclude autofluorescence (Figure S1). Fluoromount^TM^ (Diagnostic BioSystems Inc., Pleasanton, CA, USA) was used to mount the coverslips on the sections to prevent photobleaching. Qualitative analysis by confocal microscopy was consistently performed by examining multiple, non-overlapping fields of view across all replicates to ensure that the observed co-localization patterns were representative and robust across all three species, and photographs were acquired using a Zeiss LSM DUO confocal laser scanning microscope equipped with a META module (Carl Zeiss MicroImaging GmbH, Jena, Germany). Two Helium-Neon (543 nm) and two argon (458 nm) lasers included in this microscope were employed, with scanning speeds of 1 min and 2 s and up to 8 averages. The images were improved using Zen 2011 (LSM 700; Zeiss Software, Oberkochen, Germany). Digital photo cropping was performed using Adobe Photoshop CC (Adobe Systems, San Jose, CA, USA) to create the figure montage [35]. We also used the ‘display profile’ function of ZEN 2011 to assess colocalization qualitatively (Figures S2–S4). Table 1 and Table 2 contain information about the primary and secondary antibodies used in this study.

### 2.6. Quantitative and Statistical Analysis

Fiji (ImageJ, 2.9.0) software was used to evaluate the intensity of the immunofluorescent staining. The quantitative analysis was performed on 10 independent fields of view. After converting the images to 8-bit, the software’s ‘Measure’ function was used to analyze the mean gray value, which evaluates the average intensity of staining in the area of interest. Higher values indicate lower intensity. Subsequently, an ANOVA was performed to examine the existence of statistically significant differences between the intensity of the immunofluorescent staining in the positive and negative controls. To quantitatively assess colocalization between markers, confocal images were first acquired under identical imaging settings to avoid variability due to acquisition parameters. Images were converted to 16-bit format and split into individual channels. Background subtraction was performed to minimize noise, and regions of interest (ROIs) were selected to ensure that analyses were restricted to representative areas of the epidermis. Colocalization analysis was then carried out using the “Coloc 2” plugin in Fiji (ImageJ), which calculates Pearson’s correlation coefficient (PCC) as a measure of the degree of pixel-by-pixel correlation between fluorescence intensities in the two channels. The analysis was repeated across multiple independent fields of view, each containing thread cells under the same experimental conditions [36]. All datasets were pre-tested for assumptions of normality (using Shapiro–Wilk test) and homoschedasticity (using Levene’s test), informing our final choice of parametric analysis.

### 2.7. In Silico Comparison of Muc2, TLR2, Vimentin and α-Tubulin Protein Domains

To detect Muc2, TLR2, Vimentin, and α-tubulin orthologs in the genome of the hagfish *Myxine glutinosa*, we used the data deposited in GeneBank (GCA_040869285.1). After identifying the orthologous proteins, we used the InterProScan v98.0 web server to functionally annotate both the reference and the newly identified *M. glutinosa* protein orthologs to describe and compare their functional domains [37].

## 3. Results

### 3.1. Histological Analysis

**Figure 1:**
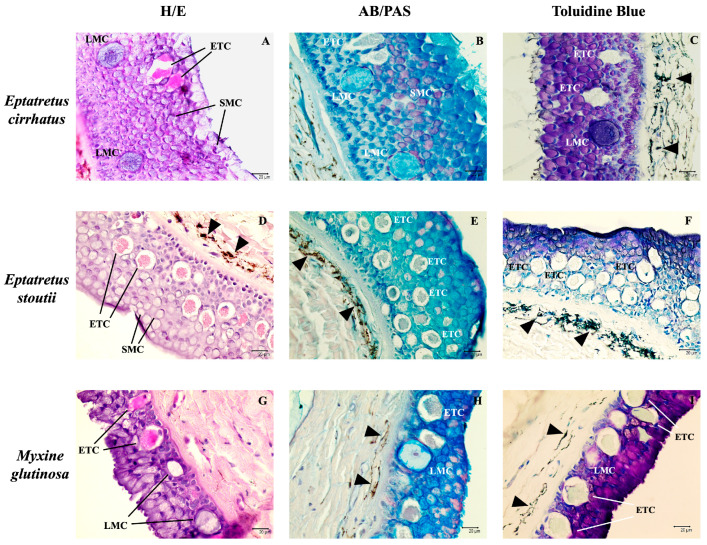
Skin cross-sections of three different hagfish species. (**A**,**D**,**G**) H/E, 40×, scale bar 20 µm. A pluristratified epithelium was evident, with many epidermal thread cells (ETCs) surrounded by many small mucous cells (SMCs). Large mucous cells (LMCs) were also observed. (**B**,**E**,**H**) Alcian blue/PAS, 40x, scale bar 20 µm. SMCs were identified based on the nature of their mucous content. LMCs appear blue because of their acidic mucous content. (**C**,**F**,**I**) Toluidine Blue, 40x, scale bar 20 µm. Furthermore, pigment cells have been observed in the dermis of (**C**–**F**,**H**,**I**) (arrowheads).

Histological examination of the skin of the three species of hagfish showed two main layers: the epidermis and dermis. The epidermis is covered by a thin non-cellular cuticle [3], and appears as a pluristratified epithelium with epidermal thread cells (ETCs) that are usually found in the middle-basal region of the epidermis, often close to large mucous cells (LMCs) (Figure 1A,D,G). They were surrounded by many small mucus cells (SMCs) (Figure 1B,E,H), identified based on the type of secretion by AB/PAS staining (neutral in magenta, acid in blue, and mixed in purple). SMCs are present in the upper portion of the epidermis, while smaller epidermal cells are layered in the basal region. ETCs show a small nucleus in the basal region of the cell, while almost the entire cytoplasm is occupied by threads that form a coiled structure and granules (Figure 1D,G). Threads are composed of intermediate filaments that act as eosinophilic structures (pink), as shown by H&E staining (Figure 1A,D,G). Instead, they were lightly stained with AB/PAS and Toluidine Blue (Figure 1), showing the presence of mucopolysaccharides and protein content, respectively. LMCs appear purple when stained with H/E or Toluidine Blue and blue when stained with AB/PAS, indicating acidic mucous content (Figure 1). Also, pigment cells were observed in the dermis (Figure 1).

### 3.2. Immunohistochemical Analysis

**Figure 2:**
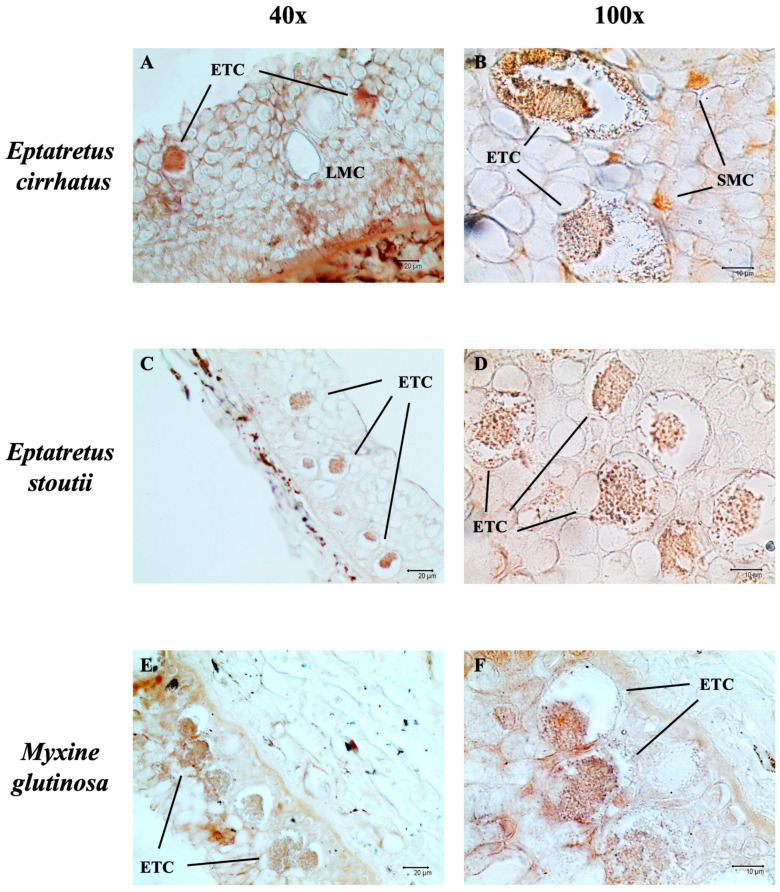
Immunoperoxidase staining of three different hagfish skin cross-sections with Piscidin-1 (brown). Scale bars: (**A**,**C**,**E**) 20 µm; (**B**,**D**,**F**) 10 µm. Legend: ETC (Epidermal Thread Cell); LMC (Large Mucous Cell); SMC (Small Mucous Cell).

Immunoperoxidase analysis revealed that the ETCs were immunoreactive for Piscidin-1 (brown color) (Figure 2). Reactivity to the tested antibody was observed in some SMCs (Figure 2B).

**Figure 3:**
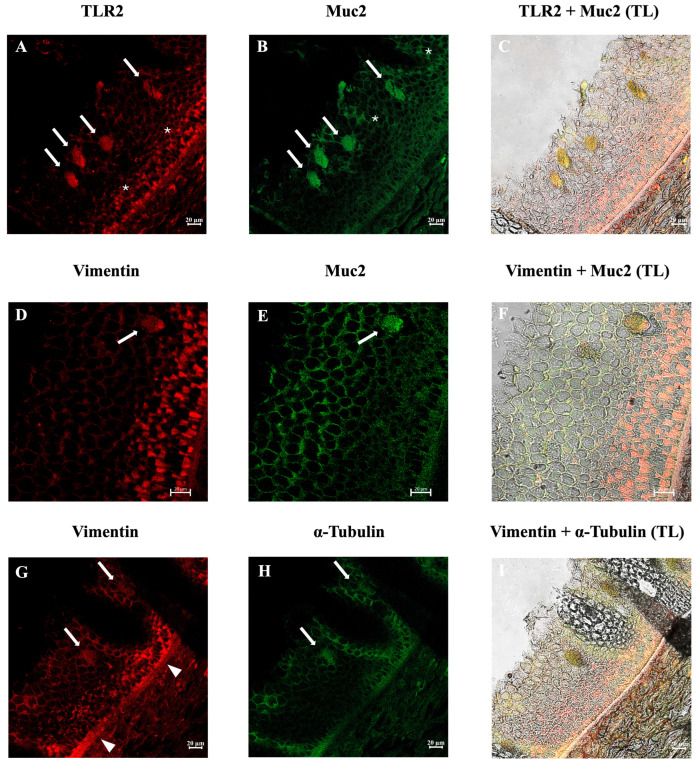
Immunofluorescence on skin cross-sections of *Eptatretus cirrhatus*. Thread cells are visible (arrows) and exhibit TLR2 (red) and Muc2 (green) immunoreactivity (**A**,**B**), with colocalization of the tested antibodies (**C**). Thread cells are immunopositive for Vimentin (red) and Muc2 (**D**,**E**), with marked colocalization (**F**). Thread cells are also immunoreactive to vimentin and α-tubulin (green) (**G**,**H**). A colocalization of these antibodies is also evident (**I**). Small mucous cells were positive for TLR2 and Muc2 (*). The lining epithelium was immunopositive for vimentin (arrowhead) and also showed a weaker stain for TLR2. Scale bars: 20 µm. (TL) Transmitted light.

**Figure 4:**
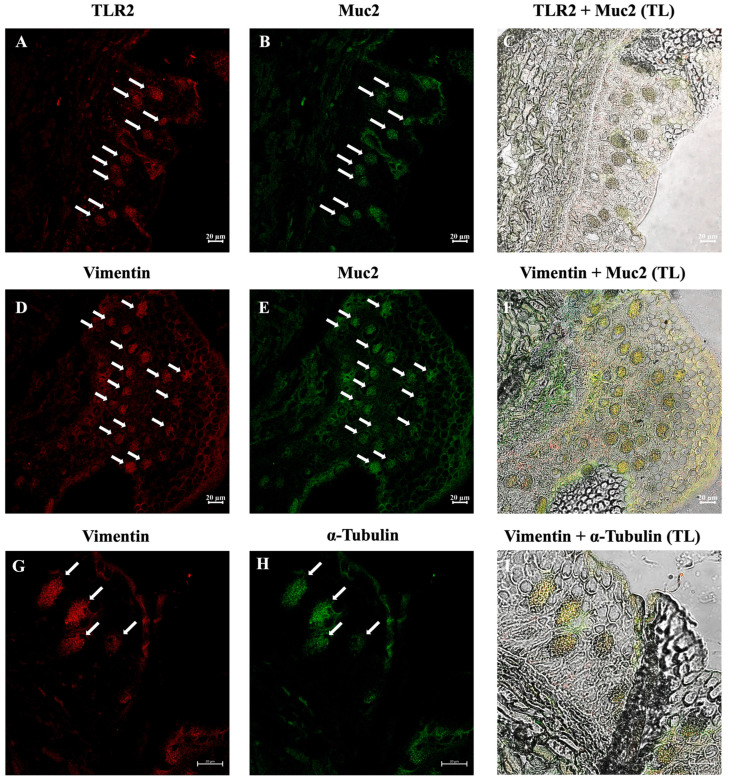
Immunofluorescence on skin cross-sections of *Eptatretus stoutii*. Thread cells are abundant (arrows) and exhibit TLR2 (red) and Muc2 (green) immunoreactivity (**A**,**B**), and the antibodies colocalize (**C**). Thread cells are also immunoreactive to Vimentin (red) and Muc2 (**D**,**E**), with a strong colocalization (**F**). The same occurs for Vimentin and α-tubulin (green) (**G**,**H**). The antibodies colocalize (**I**). Scale bars: 20 µm. (TL) Transmitted light.

**Figure 5:**
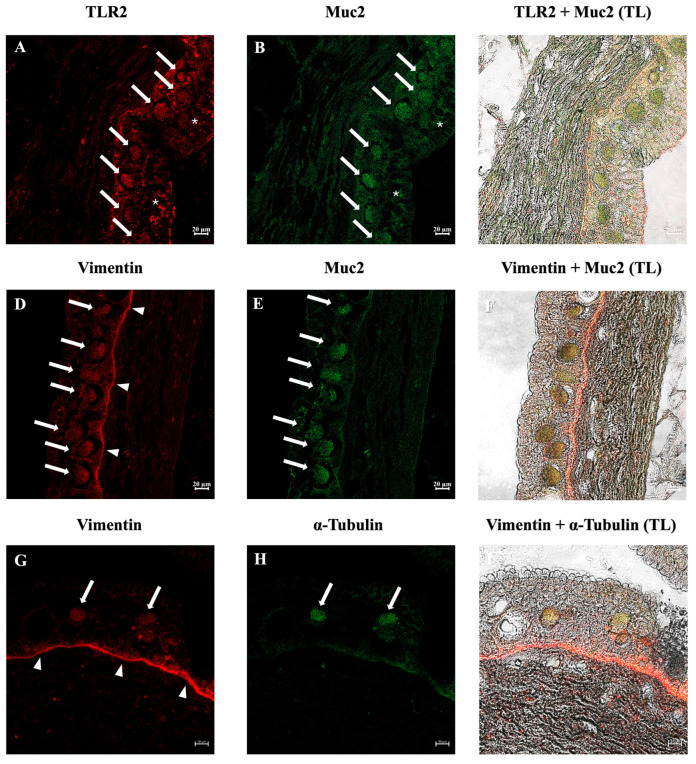
Immunofluorescence on skin cross-sections of *Myxine glutinosa*. Thread cells can be seen (arrows), they exhibit TLR2 (red) and Muc2 (green) immunoreactivity (**A**,**B**), and the antibodies colocalize (**C**). Thread cells are positive to Vimentin (red) and Muc2 (**D**,**E**), with an evident colocalization (**F**). Vimentin and α-tubulin (green) highlight thread cells (**G**,**H**). The antibodies colocalize (**I**). Small mucous cells were positive for TLR2 and Muc2 (*). The lining epithelium was immunopositive for vimentin (arrowhead). Scale bars: 20 µm. (TL) Transmitted light.

Immunofluorescence analysis revealed that many ETCs were positive for the antibodies tested in all three species examined. The three species showed similar results. Double immunolabeling with antibodies against TLR2 and Muc2 showed positive ETCs scattered in the epidermis, with colocalization of the antibodies. These cells were also immunoreactive to vimentin and α-tubulin. In addition, positivity for TLR2 and Muc2 on SMCs membranes was evident (Figure 3, Figure 4 and Figure 5). The lining epithelium was positive for Vimentin and TLR2. *E. stoutii* shows the highest abundance of thread cells in the examined epidermis sections.

Negative controls for immunofluorescence were performed on skin sections from *Eptatretus cirrhatus*, *Eptatretus stoutii*, and *Myxine glutinosa*. Incubation of the sections with the primary antibody combinations—TLR2 + Muc2, Vim + Muc2, and Vim + α-tub (Vimentin and α-tubulin)—or with the secondary antibodies alone, yielded no detectable specific signal (Figure S1).

Fluorescence intensity measurements revealed consistent patterns across the three species examined. For all marker combinations (Vim + Muc2, TLR2 + Muc2, and Vim + α-tub), mean fluorescence intensity values were lower in *E. cirrhatus*, *E. stoutii*, and *M. glutinosa* compared to their respective negative controls (Table 3).

Moreover, the statistical analysis performed revealed interesting results. The Vim + Muc2 combination exhibited substantially higher fluorescence intensity compared to controls (*t*(18) = −40.3, *p* < 0.001, Cohen’s *d* = −18.0). Similarly, the TLR2 + Muc2 combination displayed markedly enhanced fluorescence relative to controls (*t*(18) = −28.5, *p* < 0.001, Cohen’s *d* = −12.8.) Finally, the Vim + α-tub combination confirmed this pattern, with significantly higher fluorescence in experimental groups than in controls (*t*(18) = −29.7, *p* < 0.001, Cohen’s *d* = −13.3). Collectively, these results indicate a robust and consistent increment in fluorescence intensity across all tested markers in experimental groups relative to negative controls.

Pearson’s correlation coefficient (PCC) yielded a mean R value of 0.78 ± 0.09 for the colocalization of TLR2 and Muc2 across 10 independent fields of view. For the colocalization of Vimentin and Muc2, the PCC mean R value was of 0.83 ± 0.04 across 10 independent fields of view. The PCC mean R value for the colocalization between Vimentin and α-tubulin was of 0.76 ± 0.09 across 10 independent fields of view. These results indicate a consistent and robust degree of overlap between the fluorescence signals, in agreement with the qualitative observations obtained by confocal microscopy.

### 3.3. In Silico Analysis of TLR2, Vimentin, Muc2, and α-Tubulin Orthologues

**Figure 6:**
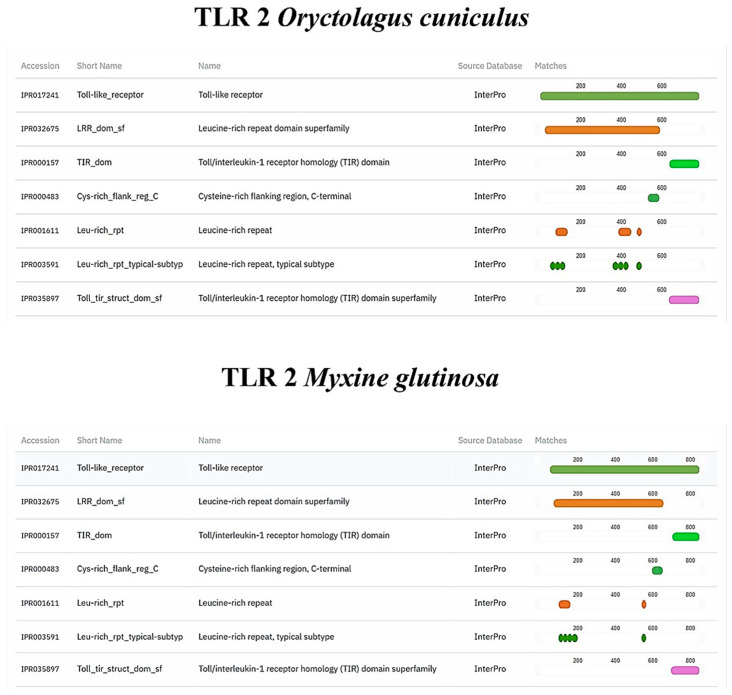
Structural and functional protein domains of *Myxine glutinosa* TLR2 ortholog and *Oryctolagus cuniculus* TLR2 protein predicted by InterProScan (v5.76-107.0).

**Figure 7:**
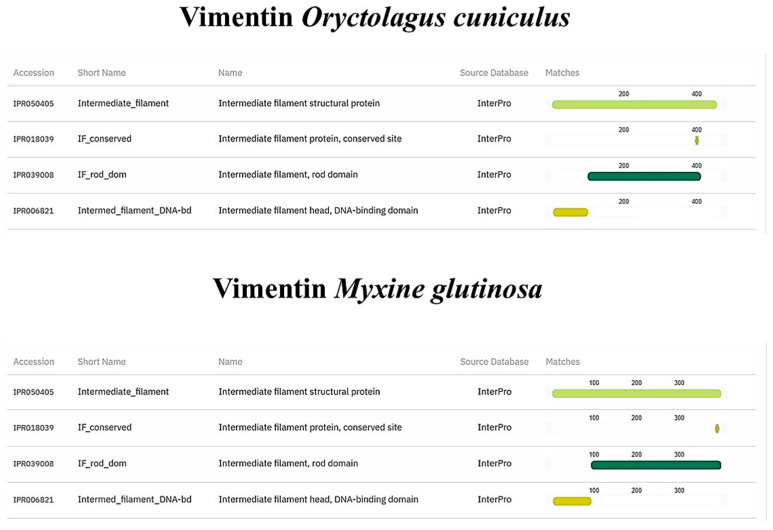
Structural and functional protein domains of *Myxine glutinosa* vimentin ortholog and *Oryctolagus cuniculus* vimentin protein predicted by InterProScan.

**Figure 8:**
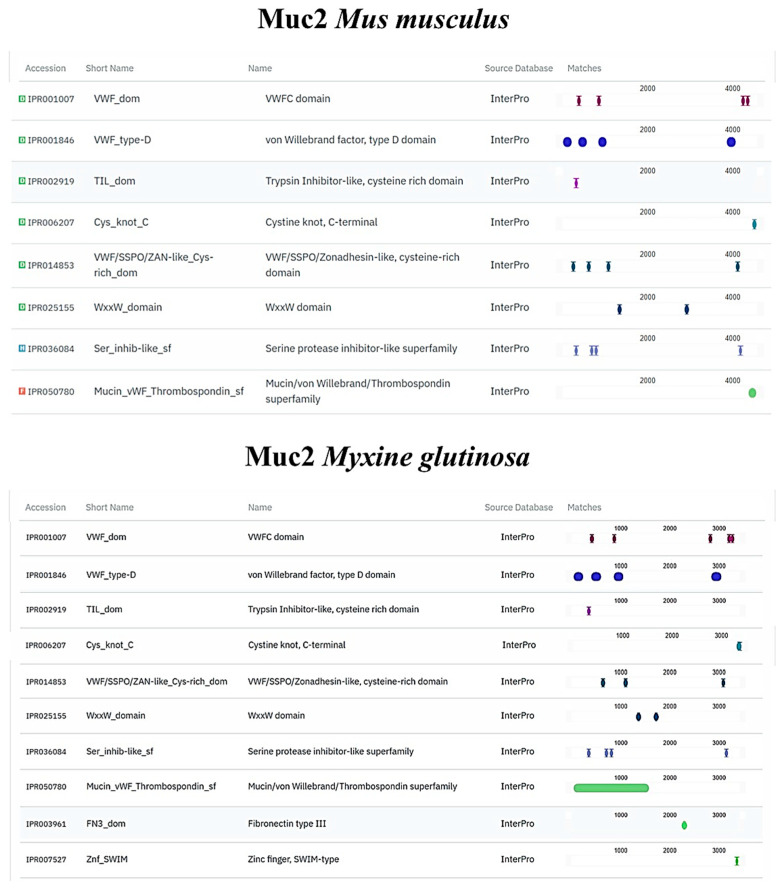
Structural and functional protein domains of *Myxine glutinosa* Muc2 ortholog and *Mus musculus* Muc2 protein predicted by InterProScan.

**Figure 9:**
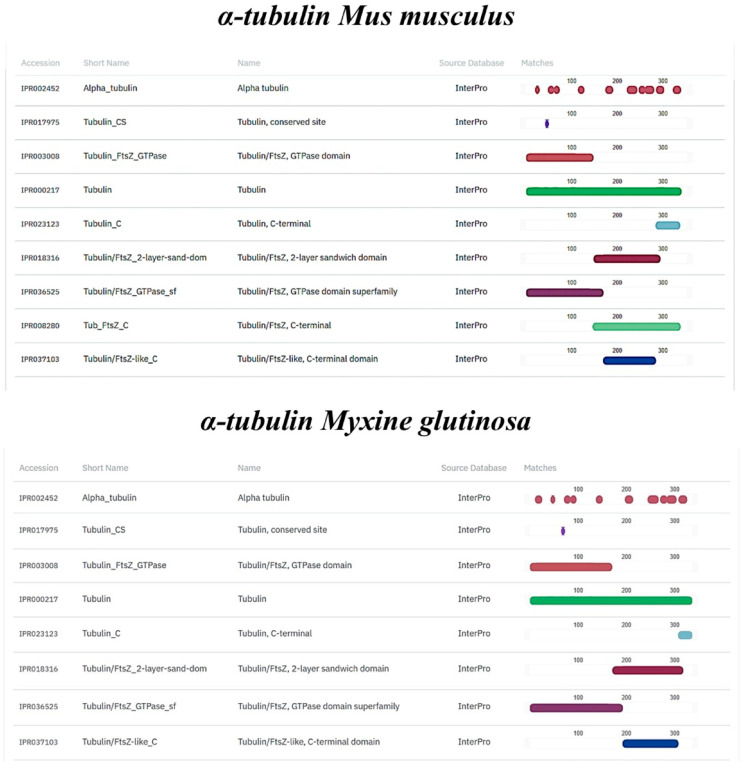
Structural and functional protein domains of *Myxine glutinosa* α-tubulin ortholog and *Mus musculus* α-tubulin protein predicted by InterProScan.

TLR2, Vimentin, Muc2, and α-tubulin orthologs were found in the *M. glutinosa* genome. Our analyses revealed the presence of one TLR2 orthologous gene that encodes an 847 aa protein. The results of the protein functional annotation step showed a significant similarity in the number and structure of the conserved protein domains. The *M. glutinosa* TLR2 ortholog and the reference TLR2 of *O. cuniculus* shared the same number and type of protein domains (Figure 6). The same similarity can be observed in the vimentin ortholog (Figure 7). Regarding the Muc2 protein ortholog prediction, we found a 3422 aa protein. The *M. glutinosa* ortholog shares eight of the ten protein domains present in *M. musculus*, with the addition of two domains exclusively found in *M. glutinosa* (IPR003961; IPR007527) (Figure 8). The α-tubulin ortholog shared eight of the nine protein domains with the reference *M. musculus*, with one domain exclusively found in *M. musculus* (IPR008280) (Figure 9).

## 4. Discussion

This study aimed to investigate the potential role of thread cells in skin defense. Usually, the main defense mechanism used by hagfish involves the release of copious amounts of slime and takes place after the hagfish gets bitten, but the low attachment of the skin to the body core allows the hagfish to survive the initial bite [38]. Slime is produced in the epidermis from specialized secretory cells: thread and mucous cells [6]. Histologically, the epidermis of all three specimens appeared rich in Small Mucous Cells (SMCs), with Epidermal Thread Cells (ETCs) and Large Mucous Cells (LMCs) usually located in the middle/basal region of the epidermis (Figure 1). Abundant SMCs rich in acidic and mixed muco-polysaccharides were revealed through histochemical AB/PAS labeling (Figure 1B). By immunohistochemical techniques with an anti-piscidin 1 antibody and confocal microscopy with antibodies against TLR2, Vimentin, Muc2, and α-tubulin, ETCs were investigated. Thread cells are unique to the epidermis of hagfish, as suggested by Fudge & Schorno (2016) [9]; their cytoplasm is almost entirely occupied by a condensed coil of intermediate filaments [11]. The release of these threads into seawater, along with mucin vesicles released from mucous cells, creates the characteristic slime [6]. The cytoplasm of thread cells is rich in granules that may be involved in defensive mechanisms and contain antimicrobial molecules [12].

Piscidin-1 belongs to a subgroup of amphipathic polypeptides that are part of the antimicrobial peptide (AMP) family, mainly found in teleosts [39]. Several studies have shown that different cell types in tissues that are in contact with the environment, such as the skin, gills, or digestive tract, are involved in the production of piscidins [23,40,41,42,43,44]. Consistent with the findings of Zeng et al. (2023) [15], the immunopositivity of thread cells for Piscidin-1 suggests that the granules could contain AMPs. We have previously demonstrated the presence of Piscidin-1 in the intestinal mucous cells of *E. cirrhatus* and its evolutionary conservation among ichthyopsids [45,46]. The results obtained in this study on Piscidin-1 immunoreactivity are consistent with the available data.

Toll-like receptors (TLRs) are Pattern Recognition Receptors involved in the modulation of the immune response and are evolutionarily conserved in all metazoans [47,48,49]. Our study showed the immunoreactivity of ETCs for TLR2, suggesting that these cells may play an essential role in the innate defense mechanisms of hagfish. Positivity for TLR2 on SMCs membranes was also evident, as shown in previous studies [50]. Following the results obtained in this study, we previously demonstrated the presence of TLR2 in the intestinal goblet cells of *E. cirrhatus*, suggesting that this molecule plays an important role in hagfish immunity [23]. Several studies have shown that the presence of TLR2 in mucous cells links mucus secretion to innate immunity, especially in the intestinal tract [51,52]. Therefore, Muc2 secretion is highly dependent on Toll-like Receptors [53]. We have described the evolutionary conservation of the sentinel role of mucous cells, finding a strong reactivity of the latter to TLR2 even in invertebrates such as *Actinia equina* (Linnaeus, 1758) [54]. The preservation of the TLR2 domain in Myxine suggests that thread cells may be capable of microbial pattern recognition (PAMPs), thus supporting your innate defense role. Furthermore, the presence of this protein in ETCs may be indicative of a distinct immune-sensing function, thus we speculate that thread cells may play a sentinel role in hagfish skin immune responses.

Vimentin is part of the type III Intermediate Filament protein family [55]. It has been demonstrated that this molecule is involved in the regulation of inflammatory responses, in cancer, and diseases of the immune system [56,57,58]. Several studies have focused on the role of vimentin in cellular functions, suggesting that this molecule exerts various effects on critical proteins involved in migration, attachment, and cell signaling [59]. Vimentin is also involved in many functions outside of cells, for example, marking circulating tumor cells and promoting neural repair [60,61]. Our results showed that ETCs were positive for vimentin, confirming the structural nature of the threads and suggesting a potential role in the defense mechanisms of these cells.

Mucins are highly glycosylated proteins produced by epithelial cells [62]. Many mucin proteins have been identified in different metazoan species, shedding light on mucin phylogenesis [63]. Gel-like mucins (Muc2) and the von Willebrand Factor share the von Willebrand D domain, which is responsible for the polymerization of mucin monomers, allowing the formation of a gel [64]. Mucins in hagfish have not been fully characterized, with only a few studies focusing on the mucous fraction of slime secretion [65]. The carbohydrate and amino acid compositions are similar to those of mucin glycoproteins, but differ in that the sulfate and proline contents are high, whereas the carbohydrate content is low [65]. The colocalization of Muc2 and TLR2 in thread cells may indicate crosstalk between mucus secretion and TLR ligands, as shown in previous research [54]. The conservation of the Muc2 domain in Myxine suggests that it is not only a structural component of the mucus but also may play a role as a key component of innate immunity at the skin level.

Tubulin is a dimeric protein involved in the formation of microtubules, cytoskeletal structures that regulate important cellular activities such as growth, division, nervous transmission, and immunity [25,66]. Tubulin subunits (α- and β-tubulin) are highly conserved among vertebrates [67]. The immunopositivity of thread cells to α-Tubulin, colocalized with Vimentin, shows a link between the cytoskeleton, microtubules, and innate immune responses, suggesting that Vimentin dynamics is dependent on microtubules, inside and outside of the cell, as shown in previous studies [68,69,70]. Because Vimentin has been shown to participate in pathogen recognition, the regulation of inflammatory pathways, and wound healing, while α-tubulin is essential for intracellular transport processes, including the trafficking of immune receptors and vesicles containing defense-related molecules, the results obtained may suggest that these proteins are structural components of the coiled intermediate filaments (threads) rather than of the granules. However, their cytoskeletal functions may indirectly contribute to the mobilization or release of immune-related molecules. The presence of these proteins in ETCs thus suggests a dual role for them, namely the maintenance of structural integrity in the threads and the potential facilitation of immune functions mediated by the cytoskeleton, such as receptor localization or secretion dynamics. The preservation of the Vimentin and α-tubulin domains may suggest that, in addition to their structural function in slime, they are involved in dynamic cellular processes such as vesicular trafficking or signaling, as occurs in the immune cells of higher vertebrates.

Our findings are consistent with those reported in the literature and provide new insights into the defense role of thread cells in the skin of Myxines. If thread cells are primarily involved in slime formation, the presence of TLR2 and Vimentin on their surface and in their cytoplasm may indicate that the organism aims to protect its internal tissues from external insults derived from its scavenger lifestyle. Although both ETCs and mucous cells were positive for Piscidin-1, TLR2, and Muc2, it is likely that their secretions differ, with mucous cells mainly producing mucins for the viscous matrix and ETCs releasing protein threads along with possible bioactive compounds from their granules. Moreover, in silico analyses of the tested antibodies strengthened the results obtained by confocal microscopy and further emphasized the evolutionary conservation of these molecules in the metazoan phylogeny.

Hagfish and lampreys, both jawless vertebrates (Cyclostomes), rely heavily on their smooth, scale-free skin for both protection and immune defense. While the hagfish utilizes a unique, complex slime structure, the lamprey, a close evolutionary relative, relies on a more ancestral system where various antimicrobial peptides (AMPs) are primarily located in mucous cells or keratinocytes of the skin epithelium [71]. Similarly, studies in teleosts show that AMPs are secreted by specific granular cells and mucous cells [72], which are directly analogous to the primary secretory cells of hagfish mucus. The striking difference, however, lies in the functional specialization observed in Myxine. The hagfish appears to have co-opted the thread cell, an entirely unique structure, to function as a highly specialized, dedicated immune effector cell capable of rapid, massive holocrine secretion. The presence of TLR2 and the capacity for cytoskeletal dynamics (Vimentin, αtubulin) in the thread cell suggests an evolutionary innovation where structural defense (the thread) and innate immune secretion (the markers we identified) are physically merged into one powerful epidermal system. This contrasts sharply with the diffuse, generalized immune response typically found across the skin epithelium of most teleosts. Our data therefore position the hagfish thread cell as a unique, highly derived immune innovation within the Cyclostome lineage, reinforcing the argument for its crucial role in the evolution of the hagfish’s survival and justifying its prominence in the defense system’s evolution.

A necessary consideration in this investigation, typical of studies involving non-model organisms, is the use of heterologous antibodies. These antibodies were raised against antigens derived from various species, and while they showed strong and specific staining patterns validated by robust controls (as detailed in the Methods), their application inherently necessitates caution concerning species cross-reactivity. Thus, while the observed localization patterns offer strong evidence for the presence and distribution of the target proteins, the precise molecular identity and binding affinity may be influenced by evolutionary divergence. Our interpretation of the cellular functions and distribution patterns, therefore, is made with this requisite level of criticality, consistent with established practice in comparative biological research. To minimize potential cross-species reactivity of the antibodies tested, in silico analyses were performed on the functional domains of the proteins under study, along with confocal microscopy observations using display profiles, positive and negative controls, fluorescence intensity measurements, and Pearson’s correlation coefficient (PCC) analysis to further confirm colocalization. All analyses confirmed the antigen–antibody interaction, supporting the hypothesis that the molecules used in this study exhibit evolutionary conservation consistent with our data.

## 5. Conclusions

In conclusion, our data provide deeper knowledge on the internal defense system of myxines. Due to the presence of specific immune markers, such as Piscidin-1 and TLR2, and of immuno-related molecules, such as Vimentin, α-tubulin, and Muc2, it can be hypothesized that thread cells may play a sentinel role in the skin innate system of hagfish.

These organisms play a significant role in research because of their unique physiology, evolutionary history, and ecological relevance, especially in deep-sea ecosystems. They are useful for studying aspects of evolution, nutrient cycling, and the health of marine ecosystems. Further research is needed to confirm the defensive role of thread cells in other hagfish species adapted to different ecosystems. However, our data may provide useful insights to help understand how these ancient vertebrates adapted to and evolved in life on the seafloor.

## Figures and Tables

**Table 1:** Antibodies data. Table reports the supplier, dilution, and animal source for each antibody used in this study.

Antibody	Supplier	Dilution µL/µL	Animal Source
Piscidin-1	GenScript, Piscataway, NJ, USA	1:200	Rabbit
TLR2	Active Motif, La Hulpe, Belgium (AB_2750977)	1:200	Rabbit
α-tubulin	Santa Cruz Biotechnology, Dallas, TX, USA (sc-23950)	1:300	Mouse, clone 6-11B-1
Muc2	Santa Cruz Biotechnology, Dallas, TX, USA (sc-515032)	1:200	Mouse, clone F-2
Vimentin	Sigma-Aldrich, St. Louis, MO, USA. (SAB1305445)	1:200	Rabbit
Alexa Fluor 488 donkey anti-mouse IgG FITC conjugated	Molecular Probes, Invitrogen, Waltham, MA, USA	1:300	Donkey
Alexa Fluor 594 donkey anti-rabbit IgG TRITC conjugated	Molecular Probes, Invitrogen, Waltham, MA, USA	1:300	Donkey

**Table 2:** Antibodies used in this study, including their supplier-reported immunogen sequences or regions, the corresponding *M. glutinosa* orthologs (NCBI accession numbers), the closest host species accession numbers, and the percentage of sequence homology.

Antibody	Immunogen Sequence/Region from the Supplier Company	Accession (NCBI)	Closest Species	Homology (%)
TLR2	a.a. 180–196, 353–370, and 473–489	XP_067975384.1	NP_036035.3	29.56
α-tubulin	15 S dynein fraction from sea urchin sperm axoneme	XP_067992527.1	NP_001019507.1	92.84
Muc2	a.a. C-terminus 4880–5179	XP_067977617.1	GAB1292918.1	33.06
Vimentin	a.a. 63–90 (includes Ser82)	XP_067959164.1	BAE29426.1	44.01

**Table 3:** Mean values ± standard deviation of fluorescence intensity for marker combinations (Vim + Muc2, TLR2 + Muc2, Vim + α-tub) in the three species (*E. cirrhatus*, *E. stoutii*, *M. glutinosa*) and their respective negative controls. Data are based on a total of N = 180 observations.

	*E. cirrhatus*	*E. stoutii*	*M. glutinosa*	Negative Control *E. cirrhatus*	Negative Control *E. stoutii*	Negative Control *M. glutinosa*
Vim + Muc2	78.358 ± 30.184	85.620 ± 32.338	77.936 ± 31.973	201.467 ± 27.482	190.938 ± 26.030	182.364 ± 25.876
TLR2 + Muc2	73.047 ± 33.332	83.576 ± 32.957	75.462 ± 42.112	179.475 ± 23.786	184.365 ± 25. 983	192.614 ± 26.572
Vim + α-tub	79.379 ± 29.981	93.355 ± 38.377	81.743 ± 37.957	187.849 ± 26.001	177.832 ± 21.374	198.583 ± 27.130

## Data Availability

The original contributions presented in this study are included in the article; further inquiries can be directed to the corresponding authors.

## Supplementary Materials

The following supporting information can be downloaded at: https://www.mdpi.com/article/10.3390/biology14121662/s1, Figure S1: Negative Control Panel for Immunofluorescence Staining. Representative images of negative controls performed on skin sections from the three cyclostomes. Scale bars: 20 µm; Figure S2: Immunofluorescence on skin cross-sections of *Eptatretus cirrhatus*, with Display profile images to visualize the colocalization. Scale bars: 20 µm; Figure S3: Immunofluorescence on skin cross-sections of *Eptatretus stoutii*, with Display profile images to visualize the colocalization. Scale bars: 20 µm; Figure S4: Immunofluorescence on skin cross-sections of *Myxine glutinosa*, with Display profile images to visualize the colocalization. Scale bars: 20 µm.
