# Supplementary material for: Allies in the Skin Defense System: The Role of Thread Cells in the Evolution of Hagfish (Myxiniformes)
Source: Biology (Basel). 2025 Nov 24;14(12):1662. doi: 10.3390/biology14121662 (PMC12729654; doi:10.3390/biology14121662)
Supplement: Supplementary file 1 [file biology-14-01662-s001.zip › biology-3940175-supplementary.pdf]

biology

Supplementary Materials

Allies in the Skin Defense System: The Role of Thread Cells in the Evolution of Hagfish (Myxiniformes)

Sebastian Marino [1,2] and Alessio Alesci [1,*]

[1] Department of Chemical, Biological, Pharmaceutical and Environmental Sciences, University of Messina, Viale Stagno d'Alcontres, 31, 98166 Messina, Italy; sebastian.marino@iusspavia.it
[2] Science, Technology and Society Class, University School for Advanced Studies IUSS, 27100 Pavia, Italy
* Correspondence: alessio.alesci@unime.it

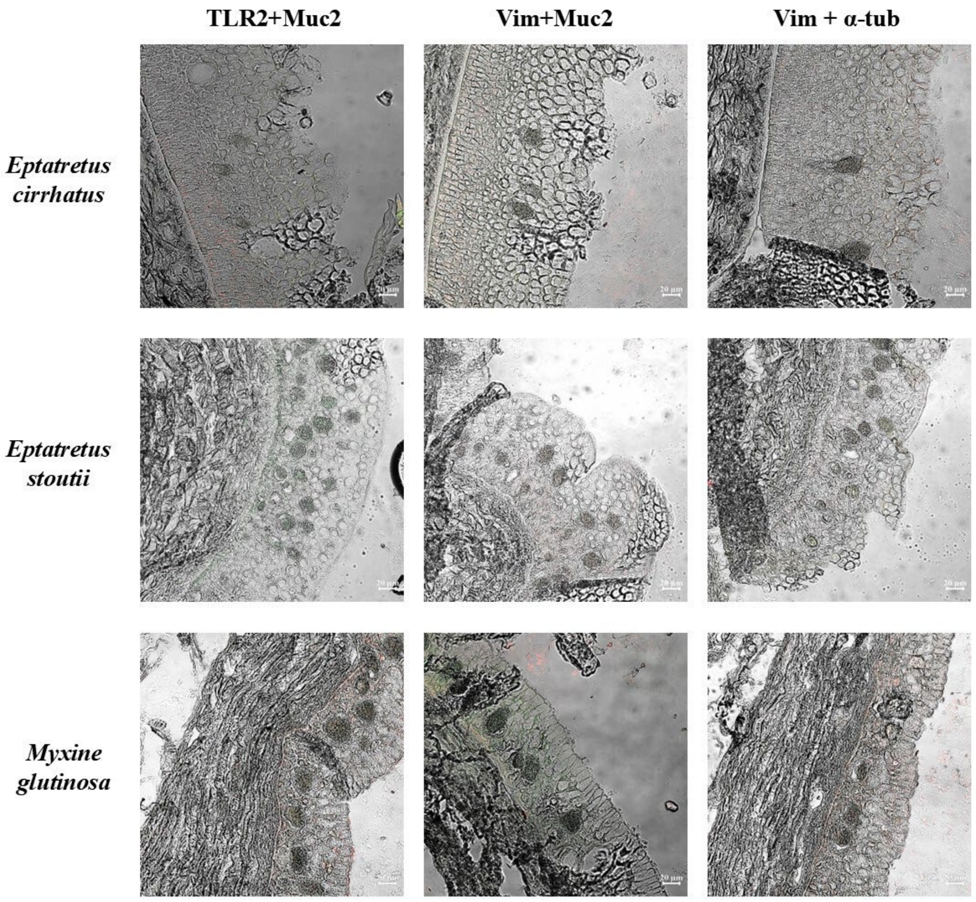

Figure S1. Negative Control Panel for Immunofluorescence Staining. Representative images of negative controls performed on skin sections from the three cyclostomes. Scale bars: 20 μm.

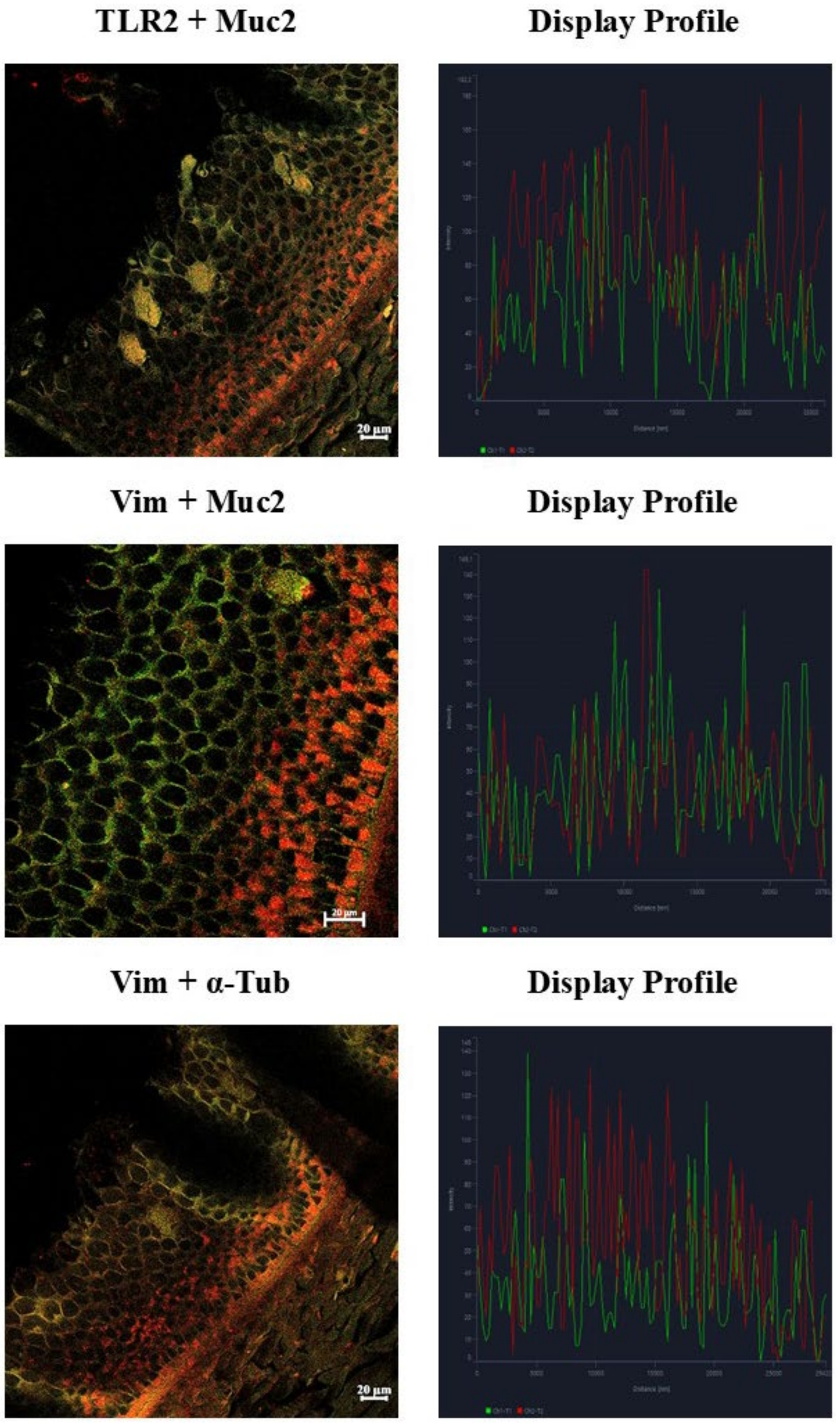

Figure S2. Immunofluorescence on skin cross-sections of *Eptatretus cirrhatus*, with Display profile images to visualize the colocalization. Scale bars: 20 µm.

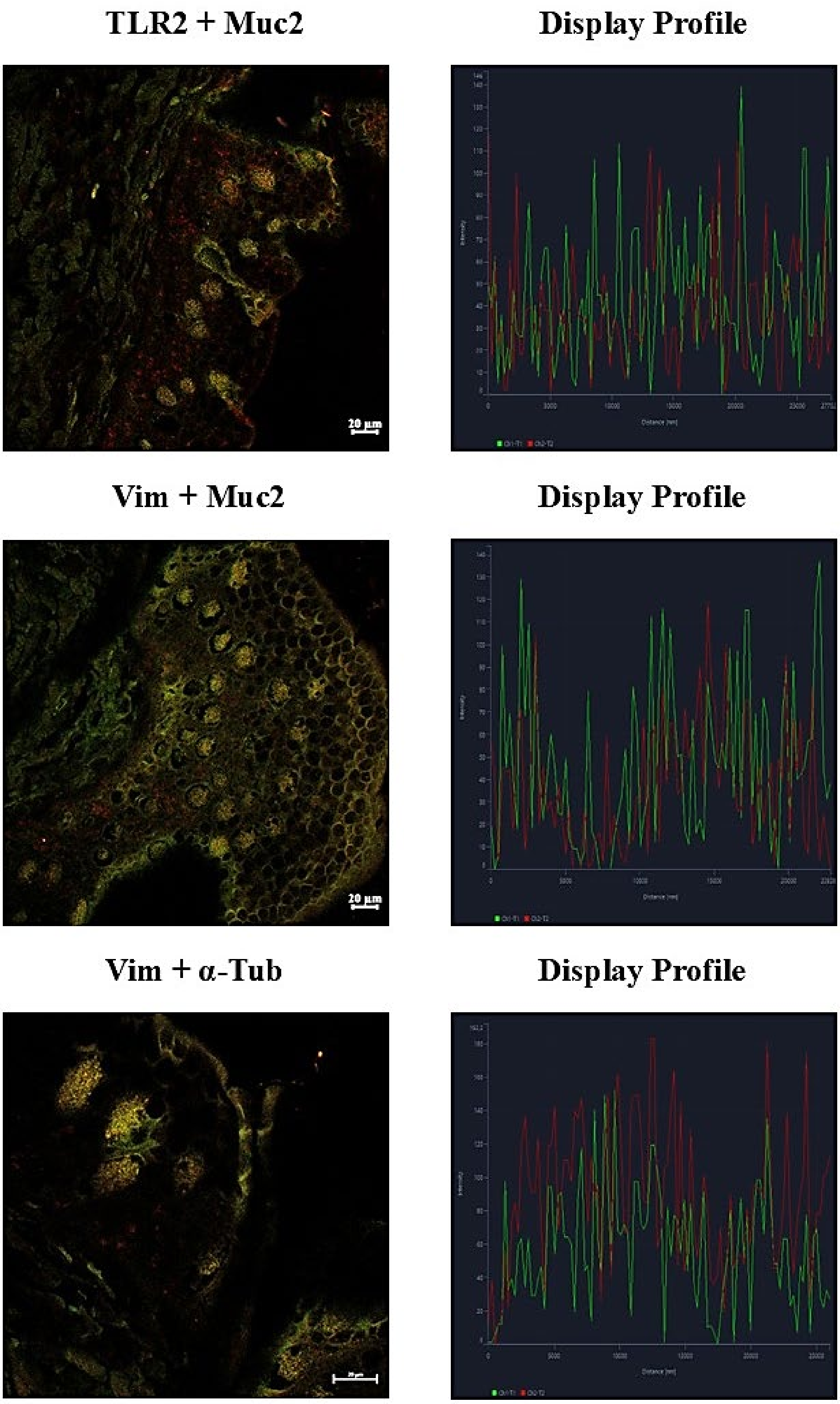

Figure S3. Immunofluorescence on skin cross-sections of *Eptatretus stoutii*, with Display profile images to visualize the colocalization. Scale bars: 20 µm.

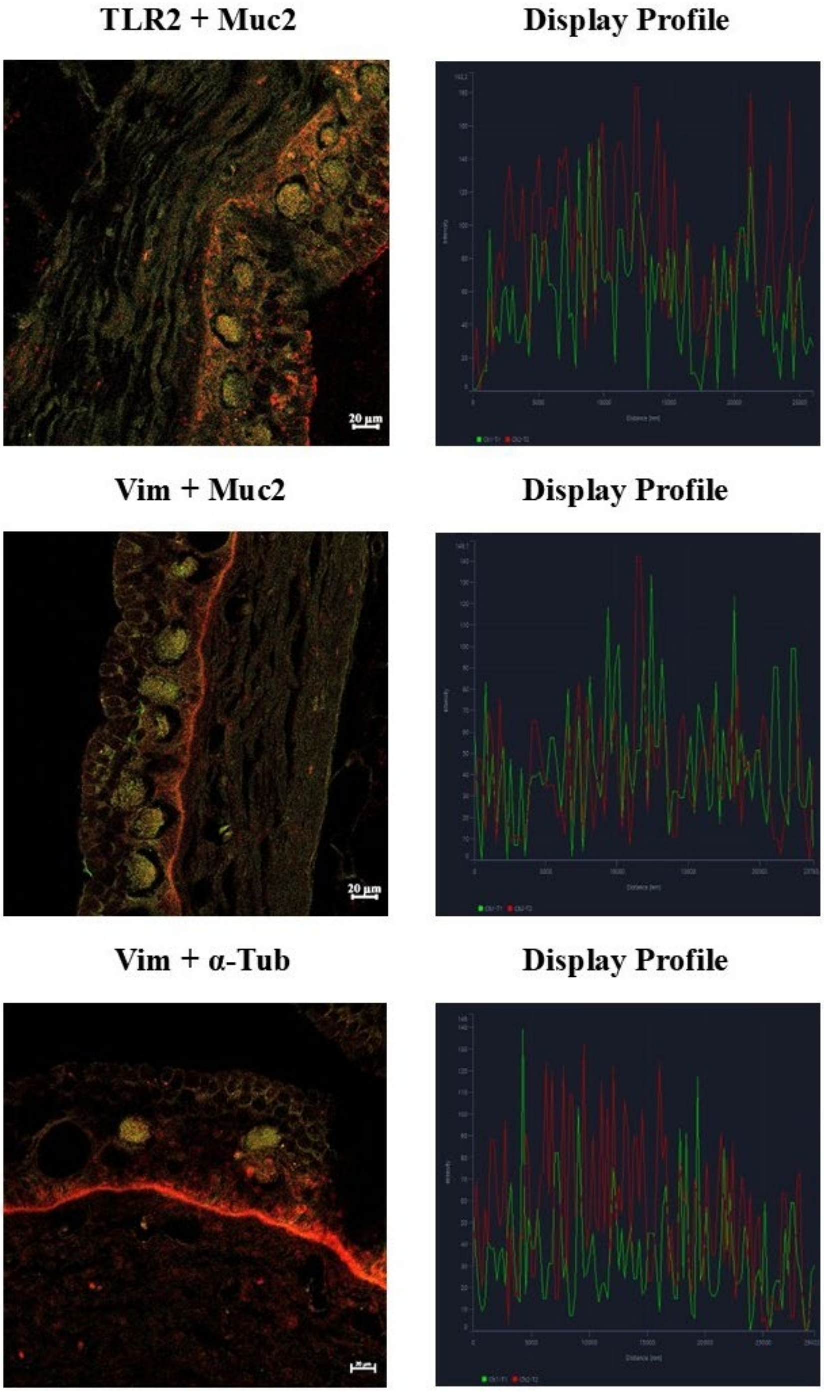

Figure S4. Immunofluorescence on skin cross-sections of Myxine glutinosa, with Display profile images to visualize the colocalization. Scale bars: 20 µm.
